# Coronary Microvascular Remodeling in Type 2 Diabetes: Synonymous With Early Aging?

**DOI:** 10.3389/fphys.2018.01463

**Published:** 2018-10-15

**Authors:** Patricia E. McCallinhart, Ian L. Sunyecz, Aaron J. Trask

**Affiliations:** ^1^Center for Cardiovascular Research, The Research Institute at Nationwide Children’s Hospital, Columbus, OH, United States; ^2^The Heart Center, Nationwide Children’s Hospital, Columbus, OH, United States; ^3^Department of Pediatrics, The Ohio State University College of Medicine, Columbus, OH, United States

**Keywords:** coronary remodeling, aging, type 2 diabetes, vascular stiffness, vascular remodeling, microcirculation

## Abstract

Type 2 diabetes mellitus (T2DM) is suggested to cause an “early vascular aging” phenomenon that is associated with vascular dysfunction, remodeling, and adverse alterations in vascular stiffness. Given that both T2DM and aging are prominent risk factors for cardiovascular disease, the aim of this study was to test the hypothesis that coronary resistance microvessel (CRM) remodeling and impairments in flow occur in the compound setting of T2DM and aging. Normal heterozygous Db/db controls and homozygous db/db mice were aged to 16 (young) or 36 (aged) weeks for all experiments and passive pressure myography and echocardiography were used to assess vascular mechanics, and structure. CRM wall thickness was significantly increased at each pressure in aged control mice compared to young control mice (9.4 ± 0.6 vs. 6.8 ± 0.2 μm, respectively, *p* < 0.001); however, there were no significant differences in CRM wall thickness of aged db/db mice vs. young db/db mice. Aged control mice had a higher medial CSA compared to young control mice (3847 ± 303 vs. 2715 ± 170 μm^2^, *p* < 0.01); however, there were no significant differences in medial CSA of aged db/db mice vs. young db/db mice. Elastic modulus was lower in aged control CRMs vs. young control CRMs (3.5x10^6^± 0.7 × 10^6^ vs. 8.7 × 10^6^± 0.6 × 10^6^, *p* < 0.0001). Elastic modulus remained the same in young db/db mice vs. aged db/db mice. These data show that the diabetic CRMs undergo adverse remodeling at an early age, similar to normal aged CRMs, that persists toward senescence, and it further suggests that diabetic CRMs are subject to an early aging phenomenon.

## Introduction

Type 2 diabetes mellitus (T2DM) is a metabolic disorder classified by the presence of hyperglycemia and insulin resistance. The American Heart Association categorizes T2DM as a cardiovascular disease in part because 2/3 of diabetes-related deaths are directly due to heart disease (Grundy et al., 1999). Myocardial infarction is 2–4 times more likely to occur in diabetic patients compared to non-diabetic patients and there is increased prevalence of coronary artery disease (CAD) in diabetic patients (Roger et al., 2011). Previously, our laboratory demonstrated that inward hypertrophic remodeling of coronary resistance microvessels (CRMs) was an early contributor to CAD and was associated with reduced coronary flow in both the db/db mouse model of type 2 diabetes and a porcine model of metabolic syndrome (MetS) (Katz et al., 2011; Trask et al., 2012).

Similarly to T2DM, aging is a prominent risk factor for cardiovascular disease (Lakatta and Levy, 2003; Hayflick, 2007). The aging myocardium undergoes adverse remodeling, including changes in composition and structure that result in cardiac dysfunction and increased left ventricular stiffness (Lakatta and Levy, 2003). The surrounding myocardium affects the structure and function of the coronary vasculature. Since aging is a comorbidity for structural cardiac changes and dysfunction, we postulate that these changes impair the coronary vasculature. In both age- and diabetes-related heart failure, remodeling of resistance vessels is a critical component of microvascular dysfunction (Gibbons and Dzau, 1994; Liu and Gutterman, 2009). Alterations in coronary arteriole structure within the heart can lead to decreased cardiac perfusion, reduced coronary flow reserve (CFR) and exacerbation of myocardial ischemia and injury. In keeping with this notion, Hanna et al. (2014) recently revealed decreased stiffness in coronary resistance arteries of aged Fisher rats, demonstrating age-related remodeling in the coronary microcirculation with age.

The (National Diabetes Statistics Report, 2017) from the CDC revealed that of the 30.3 million people with diabetes 12 million people were 65 years or older and 14.3 million people were between the ages of 45 and 64 (2017). More recently, the age of diagnosis for T2DM has been declining at an alarming rate, including increased diagnoses in the pediatric population (Hotu et al., 2004; Celik et al., 2010). Asymptomatic cardiovascular impairments such as endothelial dysfunction and adverse microvessel remodeling occur earlier in life for people with type 2 diabetes compared to non-diabetic people (Regensteiner et al., 1995; Steinberg et al., 1996; Gusso et al., 2008; From et al., 2009; Nadeau et al., 2009). The long term impact of these early, premature cardiovascular impairments in diabetes is a current area of interest for targets of clinical intervention. The goal of this present study was to test the hypothesis that CRM remodeling and impairments in flow occur in the compound setting of T2DM and aging.

## Materials and Methods

### Mouse Model

Experiments were performed on 16- (young) and 36- (aged) wk male homozygous diabetic (db/db) and age-matched heterozygous Db/db non-diabetic control mice obtained from The Jackson Laboratories (*n* = 7–11 per group; Bar Harbor, ME). The db/db mouse develops T2DM by 6–8 weeks of age as evident by obesity, hyperglycemia, insulin resistance, and dyslipidemia, therefore it is a suitable model for these studies. Mice were housed under a 12-h light/dark cycle at 22°C and 60% humidity. They were allowed *ad libitum* access to water and standard laboratory mouse low-fat chow. This study was conducted in accordance with the National Institutes of Health Guidelines, and it was approved by the Institutional Animal Care and Use Committee at Nationwide Children’s Hospital.

### Glucose Measurements

Mice were fasted for 8-h during the light cycle and blood was drawn from the tail vein. Blood glucose was measured using the AlphaTrak meter calibrated specifically for rodents (Abbot Laboratories, Alameda, CA, United States).

### Preparation of Coronary Arterioles

Mice were anesthetized using 3% isoflurane, vaporized with 100% oxygen. The heart was excised and dissected in 4°C physiologic salt solution (PSS) composed of the following (in mM): 130 NaCl, 4 KCl, 1.2 MgSO_4_, 4 NaHCO_3_, 10 HEPES, 1.2 KH_2_PO_4_, 5 glucose, and 2.5 CaCl_2_ at pH 7.4. Septal coronary arterioles (<120 μm internal diameter) at the level of the superior papillary muscle were isolated, excised and mounted onto two glass microcannulas within a pressure myograph chamber (Living Systems, Burlington, VT, United States). Prior to any measurements, vessels were equilibrated for 30 min under constant intraluminal pressure (50 mmHg) at 37°C in PSS. Internal diameter and left and right wall thickness were continuously monitored by a video image analyzer and data were recorded.

### Measurements of Coronary Arteriole Structure and Passive Mechanical Properties

Coronary resistance microvessel remodeling and mechanics were conducted as previously described by us (Katz et al., 2011; Trask et al., 2012). Briefly, experiments were performed in Ca^2+^-free PSS in the presence of 2 mM EGTA and 100 μM sodium nitroprusside. A passive pressure-diameter curve was generated by increasing intraluminal pressure from baseline (0 mmHg) up to 125 mmHg, and left and right wall thickness (WT) and internal diameters (D_i_) were recorded at each pressure. This range of pressure encompasses the physiological range in these animals *in vivo*. The following structural and mechanical parameters were calculated:

*External diameter* (D_e_) = D_i_ + (2 × WT)

*Wall/lumen ratio* = (WT/D_i_) × 100

*Cross Sectional Area* (CSA) = π (D_e_^2^–D_i_^2^)/4

*Circumferential Stress* (σ) = (*P* × *D*_i_)/(2WT), where P is pressure in dynes per square centimeter, *D*_i_ is the internal diameter for a given intraluminal pressure and WT is the wall thickness for a given intraluminal pressure.

*Circumferential Strain* (ε) = (*D*_i_–*D*_0_)/*D*_0_, where *D*_i_ is the internal diameter for a given intraluminal pressure and *D*_0_ is the reference diameter measured at 10 mmHg of intraluminal pressure.

*Young’s elastic modulus* (E) = stress (σ)/strain (ε) was used to determine arterial stiffness. However, since the stress-strain relationship is non-linear we also obtained the tangential or incremental elastic modulus (E_inc_), or simply the slope of the stress-strain relationship (i.e., Δσ/Δε).

### Coronary Blood Flow

Coronary blood flow (CBF) was measured non-invasively with a high-frequency, high-resolution ultrasound unit (Vevo2100, VisualSonics, Toronto, ON, Canada) equipped with a 30 MHz probe, at baseline and at hyperemia as previously described by us (Katz et al., 2011). Doppler measurement of the left main coronary artery diameter and flow were performed under a modified four chamber view. Mice were anesthetized with 2% isoflurane vaporized with 100% oxygen. Following induction, isoflurane was reduced to 1% to determine baseline coronary flow, and then increased to 3% to measure maximal coronary flow (Hartley et al., 2007, 2009, 2011; Wikstrom et al., 2008). We previously validated this method against adenosine for the accurate measurement of coronary blood flow and flow reserve (Katz et al., 2011). Data were analyzed offline by one observer to eliminate inter-observer variability. CBF was calculated using the equation:

*CBF* (mL/min) = [(π/4) × D^2^ × VTI × HR]/1000 where D is the internal coronary diameter (in mm) measured in B-mode ultrasound images, VTI is the velocity-time-integral (in mm), or area under the curve of the Doppler blood flow velocity tracing, and HR is heart rate.

*Coronary Flow Reserve (CFR)* = CBF_hyperemia_/CBF_baseline_ where CBF_hyperemia_ is the coronary flow measured during 3% isoflurane administration.

### Statistics

All data are represented as mean ± SEM with a probability of *p* < 0.05 used to denote statistical significance. CRM structural and biomechanical measurements and calculations were analyzed using two-way repeated measures ANOVA followed by a *post hoc* Bonferroni test. All other measurements were analyzed using an unpaired student’s *t*-test using Prism 5.0 (GraphPad, La Jolla, CA, United States).

## Results

### Body Weight and Blood Glucose

In both young and aged mice, T2DM db/db mice had significantly higher body weight and fasting blood glucose compared to heterozygous control mice (**Table 1**), verifying the presence of obesity and hyperglycemia in this model.

**Table 1:** Body weight and fasting blood glucose levels.

	Young			Aged		
	Control	db/db	*P*-value	Control	db/db	*P*-value
Body weight (g)	33 ± 1	50 ± 2	0.0001	34 ± 1	40 ± 2	0.0003
Fasting blood glucose (mg/dl)	121 ± 6	394 ± 50	0.0001	152 ± 5	635 ± 25	0.0001

*Values are mean ± SEM, n = 8 mice per group.*

### CRM Structure

**FIGURE 1:**
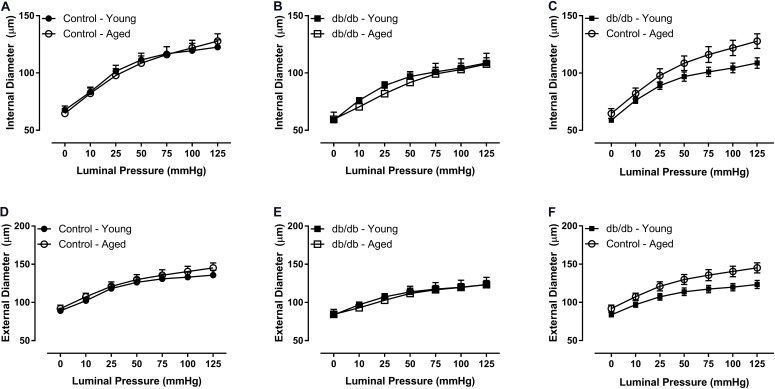
Passive diameter measurements of isolated coronary resistance microvessels (CRMs) from young and aged, normal and diabetic mice. There were no significant internal **(A–C)** or external **(D–F)** diameter differences between the age groups in CRMs from normal and diabetic mice. Values are mean ± SEM; *n* = 7–11 per group.

**FIGURE 2:**
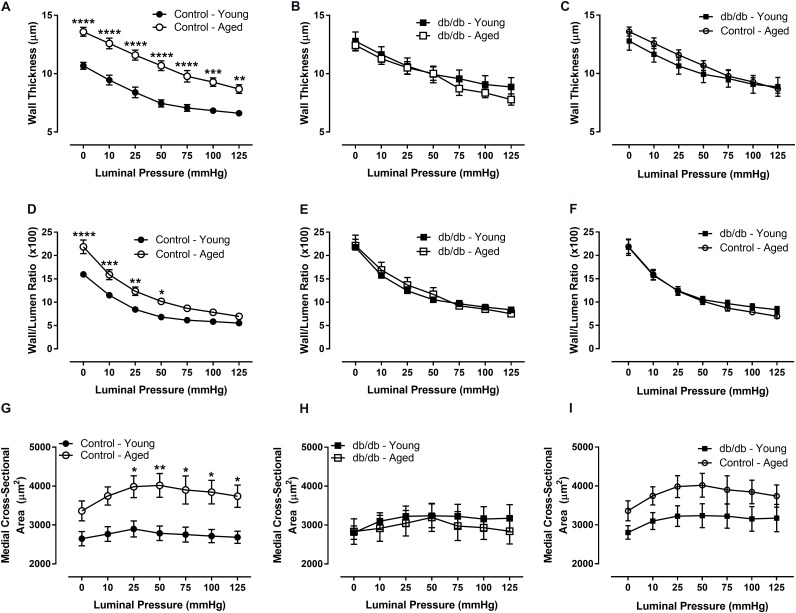
Passive structural measurements of isolated CRMs from young and aged, normal and diabetic mice. We observed increased wall thickness in the aged, control CRMs compared to young, control CRMs **(A)**, no difference in wall thickness in the diabetic mice between age groups **(B)**, no difference in wall thickness between young diabetic and aged control CRMs **(C)**, increased wall-to-lumen ratio in the aged, control CRMs compared to young, control CRMs **(D)**, no difference in wall-to-lumen ratio in the diabetic mice between age groups **(E)**, similar wall/lumen ratio between young diabetic and aged control CRMs **(F)**, increased medial cross-sectional area in the aged, control CRMs compared to young, control CRMs **(G)**, no changes in wall thickness in the diabetic mice between age groups **(H)**, or between young diabetic and aged control CRMs **(I)**. Values are mean ± SEM; *n* = 7–11 per group; ^∗^*p* < 0.05, ^∗∗^*p* < 0.01, ^∗∗∗^*p* < 0.001, and ^∗∗∗∗^*p* < 0.0001 between young vs. aged.

There were no significant internal or external diameter changes in CRMs between aged and young mice, regardless of their diabetic status (**Figure 1**). CRM wall thickness was significantly increased at each pressure in aged control mice compared to young control mice, driving a concomitant increase in wall/lumen ratio (**Figures 2A,D**); however, there were no significant differences in CRM wall thickness or wall/lumen ratio in aged diabetic mice vs. young diabetic mice (**Figures 2B,E**). Aged control mice have a higher medial CSA compared to young control mice; however, there were no significant differences in medial CSA of aged diabetic mice vs. young diabetic mice (**Figures 2G,H**). No differences existed in wall thickness, wall/lumen ratio, or mCSA between young diabetic and aged control CRMs (**Figures 2C,F,I**).

### CRM Mechanics

**FIGURE 3:**
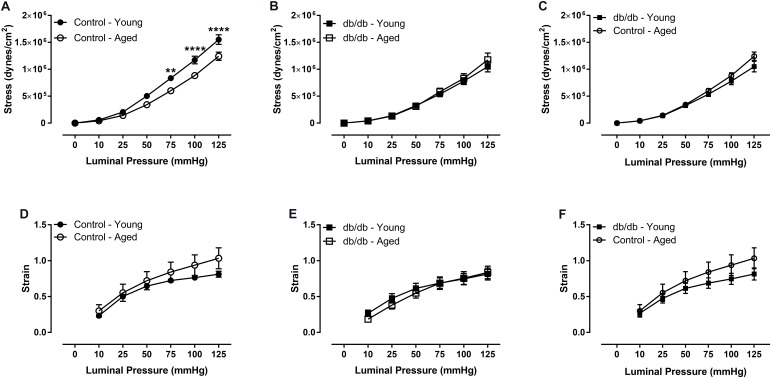
Vascular stress and strain of CRMs isolated from young and aged, normal and diabetic mice. There was a downward shift in the stress curve for CRMs isolated from the aged, control CRMs compared to young, control CRMs **(A)** and no difference in stress in the CRMs from diabetic mice between age groups **(B)**, and no difference between young diabetic and aged control CRMs **(C)**. There were no significant differences in strain between any of the groups **(D–F)**. Values are mean ± SEM; *n* = 7–11 per group; ^∗^*p* < 0.05, ^∗∗^*p* < 0.01, ^∗∗∗^*p* < 0.001, and ^∗∗∗∗^*p* < 0.0001 between young vs. aged.

**FIGURE 4:**
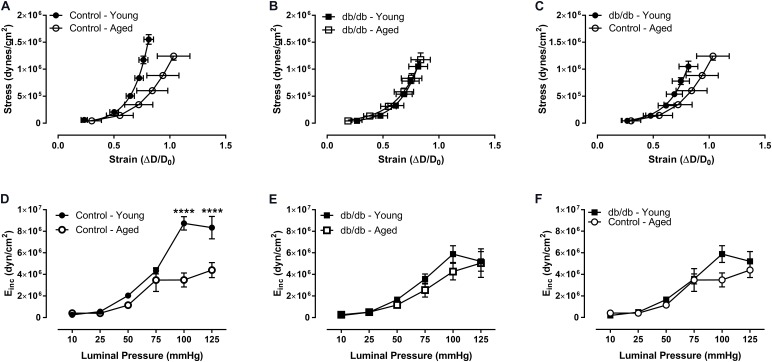
Vascular stress/strain relationship and stiffness of CRMs isolated from young and aged, normal and diabetic mice. The stress/stain curve was shifted to the right and the slope was reduced in the aged, control CRMs compared to young, control CRMs, indicating decreased vascular wall stiffness **(A)**, and there were no changes in the CRM stress/stain curve in the diabetic mice between age groups **(B)**, nor between young diabetic and aged control CRMs **(C)**. Correspondingly, incremental elastic modulus was significantly reduced in the aged, control CRMs compared to young, control CRMs **(D)** and there were no changes in the CRM stiffness in the diabetic mice between age groups **(E)**, nor between young diabetic and aged control CRMs **(F)**. Values are mean ± SEM; *n* = 7–11 per group; ^∗∗∗∗^*p* < 0.0001 between young vs. aged.

We assessed CRM wall mechanics by determining stress, strain, and incremental modulus (E_inc_) to ascertain vessel stiffness. Stress was reduced at physiological pressures in aged control mice (**Figure 3**), which drove a rightward shift and slope reduction in the stress-strain curve of CRMs from aged control mice compared to CRMs from young control mice, indicating decreased CRM stiffness with age (**Figure 4**). There were no significant differences in stress, strain, or the stress-strain curve in CRMs of the aged vs. young diabetic mice, nor in the young diabetic vs. aged control mice (**Figures 3C,F**, **4C,F**). Calculations of E_inc_, a geometry-independent measurement of vessel stiffness, corroborated the significant reduction in CRM stiffness in the aged control mice vs. young control mice (**Figure 4D**). E_inc_ remained the same in young diabetic mice vs. aged diabetic mice and between young diabetic mice and aged control mice (**Figures 4E,F**).

### Coronary Blood Flow

**FIGURE 5:**
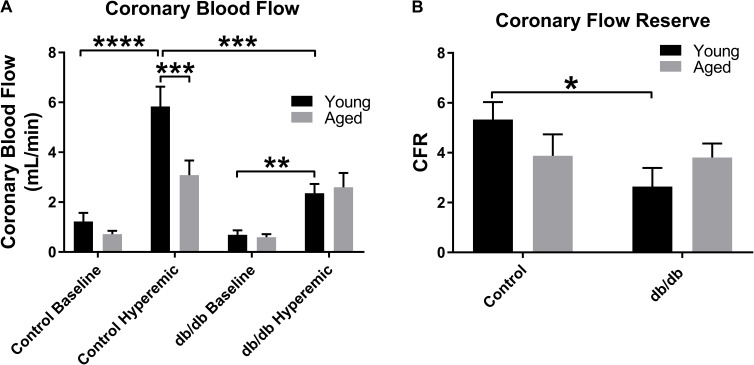
Baseline and hyperemic coronary blood flow and coronary flow reserve (CFR) in young and aged, normal and diabetic mice. CBF trended downward at baseline and was significantly decreased during hyperemia in aged control mice vs. young control mice to a similar degree as observed in the diabetic mice **(A)**. There were no significant differences between the two age groups in the diabetic mice. As we have shown previously, CFR was reduced in young db/db mice vs. young control mice **(B)**; however, we did not observe any alterations in CFR with age. Values are mean ± SEM; *n* = 4–8 per group; ^∗^*p* < 0.05, ^∗∗^*p* < 0.01, ^∗∗∗^*p* < 0.001, and ^∗∗∗∗^*p* < 0.0001 where indicated.

Coronary blood flow was measured at baseline (1% isoflurane) and hyperemic-conditions (3% isoflurane). As in previous studies by our group, we confirm here a reduction in CBF between young normal and diabetic mice (**Figure 5A**). With respect to aging, CBF trended downward at baseline (*p* = 0.12) and was significantly decreased during hyperemia in aged control mice vs. young control mice (**Figure 5A**). There were no significant differences between the two age groups in the diabetic mice. As we have shown previously, CFR was reduced in young db/db mice vs. young control mice (**Figure 5B**); however, we did not observe any alterations in CFR with age, likely driven by a reduction in hyperemic CBF in aged control mice (**Figure 5A**). Heart rates and coronary diameters at the time of coronary Doppler echocardiography are shown in **Table 2**.

**Table 2:** Heart rates and coronary diameters measured at the time of coronary Doppler echocardiography.

	Young			Aged		
HR	Control	db/db	*P*-value	Control	db/db	*P*-value
*Baseline*	377 ± 15	371 ± 13	0.75	395 ± 22	346 ± 9	0.08
*Hyperemia*	442 ± 14	364 ± 6	0.002	429 ± 13	335 ± 13	0.0002
**Coronary diameters (μm)**						
*Baseline*	416 ± 34	302 ± 25	0.04	337 ± 20	331 ± 28	0.86
*Hyperemia*	561 ± 22	442 ± 26	0.01	428 ± 52	445 ± 38	0.80

*Values are mean ± SEM, n = 4–8 mice per group.*

## Discussion

Our lab has previously revealed that T2DM leads to premature cardiovascular impairments including coronary microvessel inward remodeling (Katz et al., 2011; Trask et al., 2012). The long term effect of early diabetic cardiovascular impairments and how the aging process further impacts these complications is poorly understood. In our current study, we examined how the early aging process influences CRM remodeling in the setting of diabetes. To accomplish this aim, we measured vessel remodeling and mechanics via pressure myography and coronary blood flow by echocardiography. Upon initial analysis, we saw no significant difference in CRM remodeling and mechanics between aged control mice vs. aged diabetic mice. We found this data to be quite intriguing, and upon analysis comparing young and aged control mice, we determined that CRMs from aged control mice underwent adverse remodeling similar to that of the young diabetic mice. Interestingly, the adverse CRM remodeling that was observed in young diabetic mice did not worsen with age, which supports the idea that T2DM is accelerating the coronary microvascular aging process.

It is well-established that during the aging process, large conduit arteries, including the aorta, have increased wall thickness and pulse wave velocity, the conventional measurement of large artery stiffness (Spina et al., 1983; O’Rourke, 1990; Kohn et al., 2015). Large vessel stiffening is part of the dogmatic aging progression due to the decline of elastic properties (Zieman et al., 2005; Greenwald, 2007; Mitchell et al., 2007; Sawabe, 2010; Cecelja and Chowienczyk, 2012). A consequence of the age-induced adverse vessel remodeling is hemodynamic instability, which most commonly manifests as a result of hypertension and anesthetic-induced hypotension resulting in reduced end-organ perfusion (Gragasin et al., 2012). Along with vessel dilation and stiffening, endothelial cell senescence is frequently associated with vascular aging (Dantas et al., 2012). Contrary to larger vessels, CRMs decrease in stiffness with age in Fisher rats (Hanna et al., 2014), Here, we show that this decrease in stiffness occurs in aged mice and surprisingly is not exacerbated by diabetes. Our data support an early peak then plateau of diabetes-induced coronary microvessel adverse remodeling that mimics age-related CRM remodeling.

In most vessels, diabetes leads to an increase in adverse remodeling and stiffening. Previous studies have shown that diabetes alters ECM structure in macrovessels including the aorta and the carotid artery, leading to vessel wall stiffening (Wolffenbuttel et al., 1998; Reddy, 2004; Marsh et al., 2009). Our lab has previously reported increased stiffness in diabetic femoral arteries (Katz et al., 2011). However, we have also reported that diabetic CRMs are less stiff and undergo inward hypertrophic remodeling in both the db/db mice and MetS Ossabaw pig models (Katz et al., 2011; Trask et al., 2012). In our current study, we show that aging does not exacerbate diabetic CRM remodeling for reasons that are currently unclear. Perhaps there is a structural limit to remodeling that occurs earlier in the diabetic condition and plateaus until senescence. We found no significant differences between young and aged diabetic CRM wall structure or mechanics; however, we did find significant differences between the age groups of the control CRMs, including decreased stiffness and wall thickening.

T2DM and aging result in many of the same co-morbidities and cardiovascular complications, causing speculation that diabetes is a form of advanced aging on the cardiovascular system. Both the aged myocardium and diabetic myocardium are much stiffer than a healthy, young myocardium (Wu et al., 1977; Woodiwiss et al., 1996; Lakatta and Levy, 2003; Ouzounian et al., 2008). One potential explanation for the decrease CRM stiffness in both conditions may be due to a compensation for the stiffer surrounding environment. Furthermore T2DM and aging lead to an accumulation of AGEs, advanced end glycation products, which could also impact the remodeling of the CRMs. Activation of RAGE, the receptor for advanced glycation end products, leads to a multitude of cellular signaling events including a robust inflammatory response. The accumulation of AGEs and RAGE during both aging and diabetes impacts cardiovascular function through the inflammatory response and AGEs contribute to a variety of microvascular and macrovascular complications via molecular cross-linking in the basement membrane of the ECM (Soulis et al., 1997; Kislinger et al., 2001; Bucciarelli et al., 2002; McNulty et al., 2007). In part, these changes may account for the similar CRM remodeling in early aging and diabetes.

It is well established that inflammation increases with age and age-related diseases. The most predominant inflammatory markers that are associated with age-related chronic diseases include interleukin-6 (IL-6), C-reactive protein (CRP), and tumor necrosis factor alpha (TNF-alpha) (Singh and Newman, 2011). Oxidative stress is often associated with this aged-induced inflammation. Previous studies have shown that aged people with diabetes have increased risk for both acute and chronic microvascular and cardiovascular complications (Nakano and Ito, 2007; Kirkman et al., 2012; Assar et al., 2016). Older diabetic animals and humans exhibit higher inflammatory markers and worsened vascular function than those either aged or diabetic alone, which may suggest that chronic low-grade inflammation in aging creates a vascular environment favoring the mechanisms of vascular damage driven by diabetes (Assar et al., 2016). Interestingly though, we saw no difference in coronary microvascular mechanics between age groups when we aged the diabetic mice, although we observed differences in mechanics of the age groups of normal mice.

Finally, CRM remodeling was associated with alterations in coronary flow (**Figure 5**). Coronary flow was blunted in aged control mice under hyperemic conditions, similar to young diabetics relative to young controls, and it trended downward at baseline, although this did not result in statistically significant reduction in CFR in aged control mice. In fact, the baseline and hyperemic coronary blood flows were very similar between the young diabetic and aged control mice, leading one to believe that the similarities in remodeling were driving the blunting of coronary flow. Given that many others have demonstrated dysfunction in coronary microcirculation with aging and diabetes, the sum total of coronary flow reduction in both early diabetes and normal aging may be due to both structural and functional deficits.

This is first study to evaluate the impact of aging in coronary microvascular remodeling and stiffness compounded with T2DM. We show that the normal coronary microcirculation undergoes adverse remodeling with age, similar to the diabetic coronary microcirculation at a much younger time point. These data suggest that T2DM imparts an accelerated early coronary microvascular aging process that is sustained with age.

## Author Contributions

PM and AT conceived and designed the experiments. PM, IS, and AT analyzed the data and contributed to the writing of the manuscript.

## Conflict of Interest Statement

The authors declare that the research was conducted in the absence of any commercial or financial relationships that could be construed as a potential conflict of interest. The reviewer SB and handling Editor declared their shared affiliation.
